# New Structural and Single Nucleotide Mutations in Type I and Type II Collagens in Taiwanese Children With Type I and Type II Collagenopathies

**DOI:** 10.3389/fgene.2021.594285

**Published:** 2021-07-28

**Authors:** Meng-Che Tsai, Yen-Yin Chou, Chia-Yi Li, Yi-Chieh Wang, Hui-Wen Yu, Chia-Hsiang Chen, Peng-Chieh Chen

**Affiliations:** ^1^Institute of Clinical Medicine, College of Medicine, National Cheng Kung University, Tainan, Taiwan; ^2^Depatment of Pediatrics, College of Medicine, National Cheng Kung University Hospital, National Cheng Kung University, Tainan, Taiwan; ^3^Research Center of Clinical Medicine, College of Medicine, National Cheng Kung University Hospital, National Cheng Kung University, Tainan, Taiwan

**Keywords:** osteogenesis imperfecta, spondyloepiphyseal dysplasia congenita, collagen, osteoporosis, next-generation sequencing

## Abstract

Collagenopathy is a rare genetic condition characterized by abnormality in either collagen structure or metabolism. Variations in its clinical presentations highlight diversity in the genetic causes and potential existence of concurrent mutations. Through whole exome sequencing (WES) complemented with multiplex ligation-dependent probe amplification, we identified the genetic etiologies for six cases with osteogenesis imperfecta (OI) in *COL1A1* (p.T1298N, p.Q1280Pfs^∗^51, and p.G557Vfs^∗^23) and *COL1A2* (c.1-1677_133-441del) as well as three cases with spondyloepiphyseal dysplasia congenita in *COL2A1* (p.G1041S, p.G654S, and p.G441A). Co-occurrence of *COL1A1* and *WNT1* mutations was found in a patient with a mild OI phenotype but severe osteoporosis. These findings extended the pathogenic variant spectrum of *COL1A1*, *COL1A2*, and *COL2A1* for type I and type II collagenopathies. Although WES provides a fast and accurate method to identify the genetic causes in most of the patients with type I and type II collagenopathies, its limitation of detecting CNVs because of variable capturing uniformity should be kept in mind when interpreting the results. Taken together, we demonstrate that multiple genetic characterizing technologies can provide an accurate and efficient molecular diagnostic of new genetic variants in disease-causing genes that are compatible with clinical phenotypes.

## Introduction

Collagenopathy is a group of disorders characterized by molecular abnormality either in the structure or metabolism of collagen proteins. Its clinical findings vary widely in affected tissue types and severity according to involved collagens and mutations (Carter and Raggio, 2009). Because collagen is the most abundant structural connective tissue protein with a particularly high expression in bones and cartilages, skeletal dysplasia becomes a prominent presentation associated with defective collagen proteins (Hall, 2002). Osteogenesis imperfecta (OI) is one of the most common genetic conditions in pediatric primary collagen disease with a rare incidence estimated around 1 in 15,000–20,000 live births (Kuurila et al., 2002; Forlino and Marini, 2016). OI is an inherited connective tissue disorder due to molecular dysfunctions in type I collagen synthesis and it manifests a wide-ranging spectrum of skeletal and non-skeletal phenotypes (Forlino et al., 2011). The clinical features in skeletal dysplasia are primarily related to bone fragility and deformity, such as macrocephaly, abnormal tooth development, flat midface and triangular facies, chest wall deformities, and scoliosis or kyphosis. It also presents non-skeletal features, including blue/gray sclerae, hearing loss, decreased pulmonary function, and cardiac valvular regurgitation.

OI cases are associated with dominant, recessive or X-linked mutations in a number of genes involved in different aspects of defects in collagen synthesis, structure or processing (*COL1A1*, *COL1A2*, *BMP1*); or defects in post-translational modification (*PLOD2*, *CRTAP*, *LEPRE1*, *P3H1*, *PPIB*, *TMEM38B*); folding or crosslinking defects (*SERPINH1, FKBP10, CREB3L1, MBTPS2*); or ossification or mineralization defects (*IFITM5, SERPINF1, SPARC*); or osteoblast differentiation defects with collagen insufficiency (*WNT1,CREB3L1, SP7, SEC24D*); endoplasmic reticulum function (*KDELR2*, *MESD*); Ca^2 +^ sensitivity (*PLS3*) and other mechanisms (*P4HB*, *P4HA1*, *TENT5A*) (Dalgleish, 1997, 1998; Morello, 2018). Obtaining genetic diagnosis with the use of conventional Sanger sequencing may not be feasible in a timely and economical manner.

Moreover, some other genetic disorders with similar skeletomuscular manifestations should be consider for differential diagnoses. For example, defective function in *COL2A1*, encoding type II collagen, may results in spondyloepiphyseal dysplasia congenita (SEDC) that is rare chondrodysplastic condition characterized by skeletal deformities such as short-trunk dwarfism, odontoid hypoplasia, cervical spine subluxation, scoliosis, kyphosis, lumbar lordosis, coxa vara, genu valgum, clubfoot, pes planus, and metaphyseal changes (Zhang et al., 2015). Conventional diagnostic process requires detailed clinical and radiographic findings, which is now increasingly confirmed through molecular genetic testing.

Herein, we presented a group of Taiwanese patients presenting skeletal dysplastic features that are compatible with primary collagen disease.

## Materials and Methods

### Subjects

A total of 9 patients diagnosed as type I and type II collagenopathies were consecutively enrolled from 7 families in this study (Supplementary Table 1). The enrollment of subjects took place in a tertiary medical center where it received pediatric referrals from a catchment area of nearly 3 million residents. The entire procedure was approved by the Institutional Review Board of the Cheng Kung University Hospital. A written informed consent was obtained from the enrollees and their guardians for the publication of the identifiable data if it applicable.

### Whole Exome Sequencing

The proband’s genomic DNA was extracted from peripheral blood collected in EDTA-containing tubes. The exome library was built using the Nextera Rapid Exome Capture kit (Illumina), which covered approximately 37 Mb of the exonic regions, to capture the fragmented genomic DNA. Pair-end sequencing 2 × 75 bp was performed on the Illumina NextSeq 500 sequencer. Sequencing reads were aligned to human genome reference Hg19 using BWA-mem^1^. Single nucleotide variants and small insertions and deletions were identified with Genome Analysis Toolkit 3.5 (GATK)^2^. Variants were annotated with ANNOVAR^3^ and novel variants were filtered against 1000 Genomes, dbSNP, and Genome Aggregation Database^4^. Variants were sorted according to the Combined Annotation Dependent Depletion (CADD) score (Rentzsch et al., 2019), and the functional effects of these amino acid substitutions were predicted using *in silico* analysis tools, such as PolyPhen 2^5^, PROVEAN^6^, SIFT^7^, MutationTaster^8^, and varSEAK^9^ (Supplementary Table 1). Segregation analysis with Sanger sequencing on the DNA of the probands and their family members was finally used to validate the potentially pathogenic variants.

### RNA Sequencing

Peripheral blood mononuclear cells (PBMCs) were isolated from 5 ml of blood using Ficoll-Paque PLUS. Blood samples from the same family were collected and processed to the library construction at the same time. Total RNA was extracted from the same amount of PBMCs using RNeasy Micro Kit (QIAGEN). Sequencing libraries from mRNA was prepared with SMART-Seq v4 Ultra Low Input RNA Kit for Sequencing (Takara Bio USA) and Nextera XT DNA Library Prep Kit (Illumina), and then sequenced with Illumina NextSeq 500 sequencer with 2 × 75 pair-end sequencing reaction with Illumina NextSeq500 system. On average, 39.46 million reads were obtained for each sample. Reads were aligned with GRCh38 using HISAT2^10^ and the aligned reads were counted with HTSeq^11^ and annotation GTF file from ENSEMBL (Homo_sapiens.GRCh38.99). Genes with fewer than 10 reads in all genotypes were not included in further analyses. Differential expression of genes in probands when compared with control was analyzed with DESeq2^12^. For gene set enrichment analysis (GSEA), normalized expression level of genes was analyzed with gene set collections from the Molecular Signatures Database v7.0^13^.

### Quantitative Real-Time Polymerase Chain Reaction

Probands with no variants detected with WES were subjected for multiplex ligation-dependent probe amplification (MLPA) analysis (Schouten et al., 2002; van Dijk et al., 2010). SYBR Green PCR Master Mix (Thermo Fisher Scientific) and primers (Supplementary Table 2) designed to detect copy number changes of *COL1A2* exon 1 and exon 4 were used to confirm the results of MLPA. Concentration of genomic DNA was measured by Qubit dsDNA BR Assay (Thermo Fisher Scientific). All reaction sets were conducted in triplicate, and a non-template control was run simultaneously. The amount of *COL1A2* was normalized to the genomic *GAPDH* and quantified using the comparative C_T_ method (ddC_T_).

### Polymerase Chain Reaction

The concentration of genomic DNA was quantified by Qubit dsDNA BR Assay Kit before used. Each reaction mix consisted of 2.5 μl of genomic DNA (150 ng), 0.5 μl of PfuUltra II Fusion HS DNA Polymerase (Agilent), 0.5 μl of dNTP (25 mM), 5 μl of 10X PfuUltra II reaction buffer, 1 μl of forward primer (10 μM), 1 μl of reverse primer (10 μM), and 14.5 μl nuclease- free water in a total volume of 25 μl. PCR was performed using Veriti^TM^ Thermal Cycler (Applied Biosystems) and was run on the program 95°C for 2 m followed by 40 cycles of 20 s at 95°C, 20 s at 57°C, and 3 m at 72°C. PCR products were visualized on 1.5% agarose gel using SYBR Safe DNA gel stain (Invitrogen) and purified by QIAquick Gel Extraction Kit (QIAGEN).

### *COL1A2* Break Point Identification and Sanger Sequencing

Gel purified-*pair 3* PCR amplicons was sequenced using the specific forward and reverse primers for break point identification (Supplementary Table 2). Sanger sequencing was performed by ABI 3730 XL DNA Analyzer (Applied Biosystems). Sanger sequencing reads were aligned to human genome reference GRCh38 to reveal the deleted region. The DNA sequence reads were performed by SnapGene Viewer.

## Results

### Familial Deletion of Exons 1–4 in *COL1A2*

**FIGURE 1:**
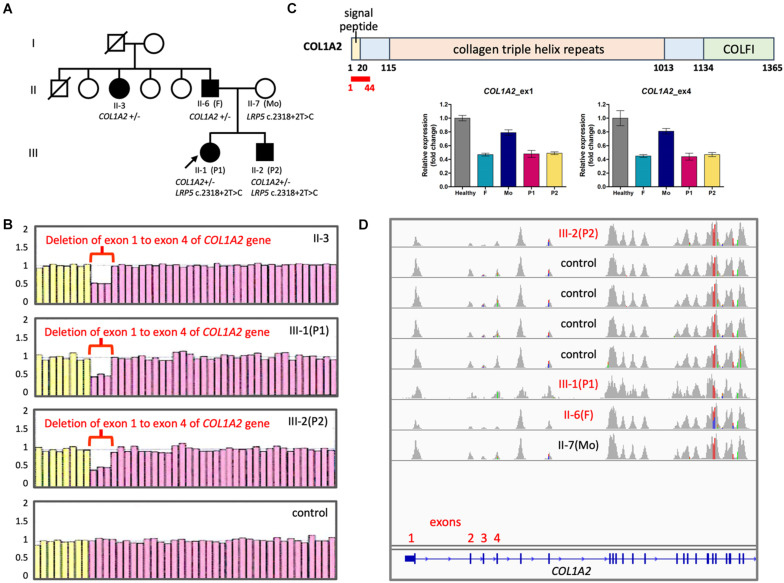
Copy number changes in *COL1A2* in the family with OI. **(A)** Family pedigree. Filled symbols, affected; half filled, heterozygous carrier; unfilled, not clinically affected. **(B)** Results of MLPA showing fold change of probes detecting *COL1A2*. Pink probes are detecting *COL1A2* while yellow probes are detecting control genes. **(C)** (Top panel) Positions of *COL1A2* exon 1–4 (encodes amino acid residue 1∼44 in red) (NP_000080.2). COLFI, Fibrillar collagen C-terminal domain. (Bottom panel) Quantification of copy numbers in *COL1A2* exon 1 and exon 4 by qPCR. **(D)** Integrative Genomics Viewer showing sashimi plots of the results of WES from OI patients and controls. Notice that read coverage of exon 2–4 of *COL1A2* are lower than other exons in general.

P1 and P2 were a pair of siblings in a family, where their father and paternal aunt also presented some mild OI phenotypes, such as blue sclera, short stature, and infrequent non-fatal fractures of extremities since childhood (Figure 1A). Their father measured 153 cm in height, which corresponded to a standard deviation score (SDS) of −4.0 as compared to average male adult height in the Taiwanese population. P1 was clinically diagnosed of OI because of blue sclera and multiple fractures in her infancy. She was wheelchair-bound since early childhood despite the use of intravenous bisphosphonate therapy. Her brother, P2, was regularly followed at the pediatric endocrinology clinic for his short stature. He had infrequent bone fractures that only required external splinting. He was found to have severe osteoporosis and hence started to receive intravenous bisphosphonate therapy, shortly after he had vertebral fractures at T9-T10 level that made him unable to walk at age 12 years.

Initially WES did not reveal pathological variants linked to OI. However, with typical phenotype of type I OI, we suspect that copy number variations (CNVs) may be the underlying cause. In order to identify potential long-range duplication or deletion in the region of *COL1A1* and *COL1A2*, the family consented for a clinical test based on MLPA technique. The results revealed loss of heterozygosity at *COL1A2* exon 1 to exon 4 that was consistently linked to the affected members within the family (Figure 1B). Quantitative PCR confirmed the copy number loss of the exons 1 and 4 (Figure 1C). Copy number change in exon 1–4 was not detected in WES because the coverage of reads was in general lower in exon 2–4 (Figure 1D).

**FIGURE 2:**
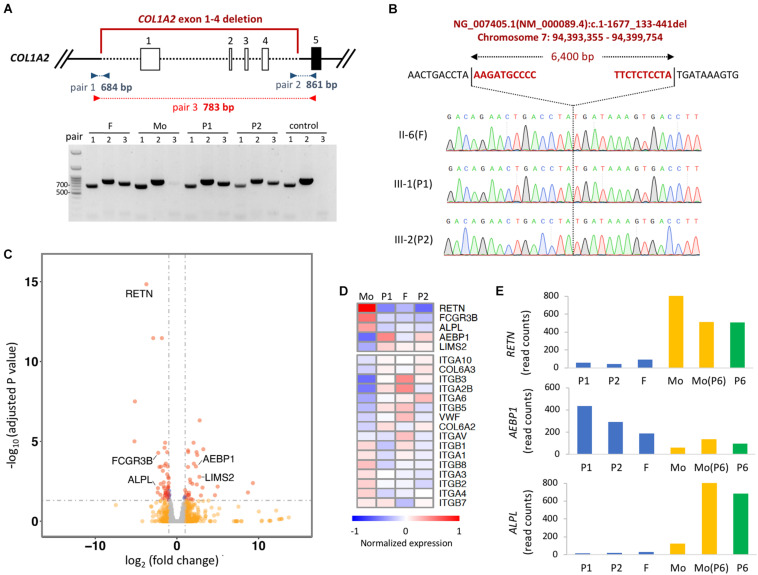
Detecting the breakpoint of heterozygous deletion in *COL1A2*. **(A)** Schematic representation of the deletion region of exon 1–4. Boxes represent exons, and dashed lined represent deleted regions. PCR amplicons used to validate the deletion with three set of primers are shown. Red primers only amplified the deleted region. **(B)** Identification of breakpoint of *COL1A2* by Sanger-sequencing the product amplified with red primers shown in **(A)**. The upper sequences show the non-deleted DNA reference sequence, while the lower sequence shows the read-out from Sanger sequencing with the breakpoint location. **(C)** Volcano plot showing differentially expressed gene in PBMCs when comparing samples with COL1A2 deletion (P1, P2, and father) vs. control (mother of P1 and P2). Red dots, genes with adjusted *P*-value < 0.05 and log_2_ fold change > 1 (*n* = 37) or < –1 (*n* = 48); blue dots, adjusted *P*-value < 0.05; orange dots: log_2_ fold change > 1 or < –1; gray dots, none of the above. **(D)** Heatmap showing DEseq2 normalized expression of genes differentially expressed when comparing P1, P2, and F vs. Mo. Mo, mother of P1 and P2; F, father of P1 and P2. **(E)** DEseq2 normalized read counts of genes in two families. Mo, mother of P1 and P2; F, father of P1 and P2; Mo (P6), mother of P6.

To identify the breakpoint of the *COL1A2* deletion, we designed multiple pairs of primer recognizing upstream of exon 1 and downstream of exon 4 to the assumptive breakpoints of the deleted segment. Polymerase chain reaction (PCR)-based electrophoresis revealed an aberrant product of 783 bp in the affected members (Figure 2A). Finally, Sanger sequencing revealed the dropped read coverage from exon 1 to 4, which located on chromosome 7 at genomic location 94,393,355–94,399,754 (GRCh38), in the *COL1A2* gene (chromosome7: 94,394,895–94,431,227) (Figure 2B).

### Coding Transcriptome in PBMC With *COL1A2* Deletion

The *COL1A2* deletion includes 1540 bp of the proximal promoter region, which is required for transcriptional regulation, and the transcription start site (Ramirez et al., 2006). Therefore, we would like to evaluate whether the expression level of *COL1A2* in the cells of the proband’s is lower than that in cells of the family members carrying normal *COL1A2*. However, because patients did not consent to tissue biopsy, we chose to evaluate the gene expression in the PBMCs isolated from their blood. Given the limited amount of RNA obtained, mRNA sequencing libraries were constructed with SMART-seq protocol and sequenced to the depth of ∼40 million reads for each sample. As expected, expression of *COL1A2* in PBMCs is extremely low (with reads aligned fewer than 10), making it difficult to compare the expression of *COL1A2* mRNA in cells with the COL1A2 deletion to the normal cells. However, when comparing the expression profile between the family members with *COL1A2* deletion (P1, P2 and father) vs. those with normal *COL1A2* (mother), we identified that 5 genes out of the 27 most differentially expressed (DE) genes (adjusted *p*-value < 0.05 and log_2_ fold change > 2 or < −2) were related to bone development or remodeling (*RETN*, *FCGR3B*, *ALPL*, *AEBP1*, and *LIMS2*) (Figures 2C,D). The trend of these differentially expressed genes are likely to be genotype-specific because of comparable expression level detected in the mother of P1 and the mother of P6 with reference allele of *COL1A2* and *COL1A1*, and P6 with *COL1A1* and *WNT1* mutation (Figure 2E). In addition, gene set enrichment analysis revealed that extracellular receptor interacting molecules, especially integrins, were also differentially expressed (Figure 2D). Taken together, these results suggest that the coding transcriptome in the circulating blood cells of patients with *COL1A2* deletion shows unique gene expression profile.

### Co-occurring Variants in Associated Genes

We observed variability in the phenotypic expression within the P1 and P2 family. While the sibling pair manifested walking disability in their childhood, their father and aunt were less affected. Therefore, we went on to investigate rare variants associated with OI that may potentially contribute to the phenotypes of the probands. Interestingly, we identified a maternally inherited splice-site variant *LRP5* c.2318 + 2T > C (Supplementary Table 3). The novel *LRP5* splice-site variant in the intron 10 was predicted to cause loss of function for 5’ splice site donor and render exon skipping as well as a premature stop codon at the beginning of exon 12. Heterozygous frameshift mutation of *LRP5* has been identified in patients with primary osteoporosis, suggesting haploinsufficiency of this gene (Hartikka et al., 2005). These results may help explain why the sibling pair had severe osteoporosis with other mild OI phenotypes.

### Co-occurring Mutations in *COL1A1*

**FIGURE 3:**
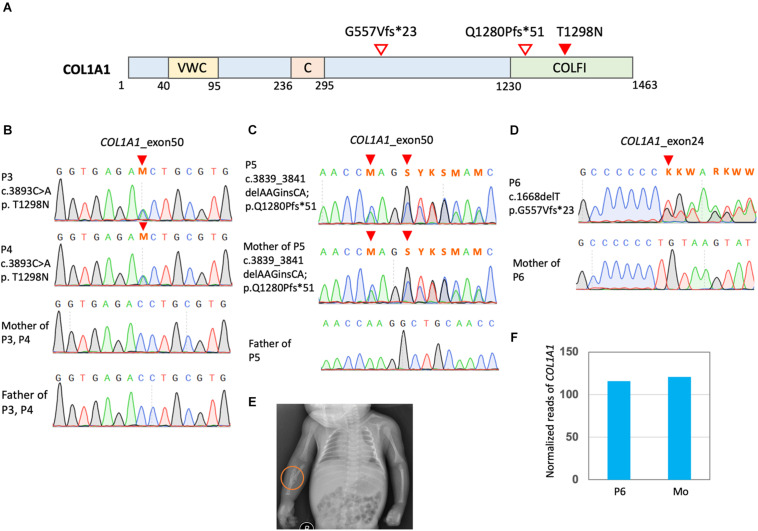
Mutation in *COL1A1* in the OI patients. **(A)** Positions of *COL1A1* (NP_000079.2) variants. VWC, von Willebrand factor type C domain; C, Collagen triple helix repeat (20 copies); COLFI, Fibrillar collagen C-terminal domain. **(B–D)** Sanger sequencing confirmed *COL1A1* mutation. **(E)** P3 presented a greenstick fracture in the mid-shaft of the right radius and ulna. **(F)** Read counts of *COL1A1* in RNA sequencing of PBMCs of OI patients P6 carrying *COL1A1* c.1668delT. Mo, mother of P6.

Four cases (P3, P4, P5, and P6) were found to have mutations in *COL1A1* (Figures 3A–D). P3 and P4 were identical twins and both had multiple perinatal fractures (Figure 3B). They also presented blue sclera and multiple fractures over their slow growth. Dual-energy x-ray absorptiometry found a decreased bone mineral density (Figure 3E). A previously known pathological mutation *COL1A1* c.3893C > A (p.T1298N) was found in these twin sisters but not in their parents (Supplementary Table 1).

P5 and her mother presented a borderline short stature with a body height at the 3rd % of the population. They also had blue sclera and several non-life-threatening fractures since birth. The mother had mild bilateral hearing impairment that did not require hearing aids. They were found to have *COL1A1* c.3839_3841delAAGinsCA; p.Q1280Pfs^∗^51 (Figure 3C). The frameshift mutations was a novel mutation and predicted to have deleterious effects (Supplementary Table 1).

P6 was found to have *COL1A1* c.1668delT (p.G557Vfs^∗^23) and a single allelic *WNT1* c.677C > T (p.S226L) (Figure 3D and Supplementary Figures 1A,B). He presented a very short stature and a kyphotic spinal curvature. He had several non-life-threatening fractures since childhood and typically blue sclera. Dual-energy x-ray absorptiometry found severe osteoporosis (Supplementary Figure 1C). The *WNT1* mutation was reported to have an allele frequency around 1.5/10,000 in the East Asian population from the gnomAD. Functionally, this mutation was predicted to be pathogenic. RNA sequencing with PBMCs detected comparable amount of *COL1A1* in P6 and his mother (Figure 3F). However, no reads containing the COL1A1 c.1668delT were detected, which is evidence of nonsense mediated decay (NMD) of the mutant transcript. On the other hand, transcripts of *WNT1* c.677C > T was detected with comparable amount of the reference allele (Supplementary Figure 1D).

### Missense Mutations in Glycine Residues in COL2A1

Three cases (P7, P8, and P9) were found to have *de novo* heterozygous mutations, *COL2A1* c.3121G > A (p.G1041S), *COL2A1* c.1960G > A (p.G654S), and *COL2A1* c.1322C > G (p.G441A), respectively (Supplementary Figures 2A–D). They all presented an extremely short stature with an abnormal spinal curvature, remarkably in the lumbar region. The bone X-ray found platyspondyly and epiphyseal dysplastic changes (Supplementary Figures 2E–G). Mutations c.1960G > A (p.G654S) and c.1322C > G (p.G441A) found in these patients were novel and predicted to have deleterious effects (Supplementary Table 1). *COL2A1* c.3121G > A (p.G1041S) has been reported to be a pathological mutation clinically associated with spondyloepimetaphyseal dysplasia (SEMD) that was also noted in P7 (Supplementary Figure 2E; Meredith et al., 2007; Zhang et al., 2015).

## Discussion

In this report, we confirmed the genetic diagnosis of 9 patients with type I and type II collagenopathies. Their clinical presentations fell into two main categories: OI (6 cases) and SEDC (3 cases). The pathogenic variants included one in *COL1A2*, four in *COL1A1*, and three in *COL2A1*. These results extended the current knowledge of genotype-phenotype correlation in type I and type II collagenopathies.

Mutations in *COL1A2* may cause symptoms ranging from severe OI to a milder Ehler-Danlos syndrome (Forlino and Marini, 2016). Less than 5% of *COL1A2* mutations can cause classic OI and they usually occur in the procollagen C-propeptide, thus impairing chain association or folding, while non-sense mutations leading to decreased synthesis of normal collagen are less detrimental (Pace et al., 2008). As compared to missense or splice-site mutations, multiexon duplication or deletion in *COL1A2* has less been reported in classic OI cases but it may be linked to a wide array of clinical OI phenotypes or several subtypes of Ehlers-Danlos syndrome (EDS), depending on the location of variant (Kuivaniemi et al., 1988; Byers et al., 1997; Raff et al., 2000). In our cases, MLPA analysis helped to complement WES by contributing toward the detection of copy number variations. Further using multiple PCR primers, we were able to identify the both breaking ends of deletion and qPCR analysis showed decreased levels of exons 1 and 4. Indeed, since the deletion in the proband occurred in exon 1, split reads with sequences with the breakpoint won’t be captured with exome-sequencing capture. The deletion of exon 1–4 was a novel structural mutation and presumed to have a deleterious effect on the functionality of mutant protein.

Although we could not obtain tissue samples from the probands, mRNA sequencing revealed DE genes related to bone homeostasis in individuals with *COL1A2* deletion. Because of similar expression level were detected in the mother of P1 and in the family with no *COL1A2* mutation, the signature of these DE genes are likely to be genotype-specific and may be the result of compensatory regulation of imbalanced level of type I α1 and α2 collagen in the cells. Further study is needed to clarify the relationship between DE genes and *COL1A2*. The expression level of these differentially expressed genes are likely to be regulated through trans-acting factors since we did not identify pathogenic mutations in these gene through WES. The most statistically significant DE gene, *RETN*, encodes for resistin, a secreted protein that regulates glucose metabolism in the cells and induces bone remodeling (Acquarone et al., 2019). In addition to circulating resistin that is produced by PBMCs, resistin is also expressed by mature osteoblasts in the bone (Acquarone et al., 2019). Moreover, resistin has been shown to stimulate osteoclast differentiation and increases osteoblast proliferation *in vitro* as well as to alter the composition of type I collagen α1/α2 ratio in human bone explants (Thommesen et al., 2006; Philp et al., 2017). Another DE gene, *ALPL*, encodes tissue non-specific alkaline phosphatase (TNAP) that is involved in mineralization of the bone (Orimo, 2010; Millan and Whyte, 2016). Loss-of-function mutations in *ALPL* may cause hypophosphatasia (HPP), a rare inherited skeletal dysplasia (Millan and Whyte, 2016). Interestingly, OI is one of the common differential diagnoses of HPP (Taillandier et al., 2015). Our finding of decreased *ALPL* expression in probands with *COL1A2* deletion may be the result of aberrant type I collagen composition, and may worsen the phenotypes of OI.

Pathogenic deletion involving the first exon of *COL1A2* (exon 1–18 deletion) has been reported in the database of collagen mutation in OI and EDS^14^. However, with the breakpoint of the deletion not determined, the expression of truncated COL1A2 protein is not clear. In our case, the deleted *COL1A2* allele is likely to produce no COL1A2. While mutations in *COL1A2* rendering premature stop codon usually have no phenotypes in heterozygote, modifying variant may contribute to the phenotypes of primary osteoporosis (Marini et al., 2007; Skarp et al., 2020). We also observed variable expressivity of the COL1A2 deletion within the family. A potential explanation is that the presence or the detrimental effect of the mutant protein varies *in vivo* within individuals or the compound variant inherited maternally aggravated the phenotypes, thus making phenotype-genotype less correlate. Functional investigation using the tissue harvested from the affected family members may be needed to verify our deduction.

Consistent with previous research, mutations in *COL1A1* gene are most likely to be reported in OI patients. We identified one known missense mutation (c.3893C > A; p.T1298N), one known frameshift mutation (c.1668delT; p.G557Vfs^∗^23), and one novel frameshift mutation (c.3839_3841delAAGinsCA; p.Q1280Pfs^∗^51) in *COL1A1* (Lu et al., 2014). Type I collagen is a heterotrimer, containing two α1 and one α2 chains. It is synthesized as a procollagen molecule, with N-terminal and C-terminal globular pro-peptides flanking the helical domain. Heterozygous *COL1A1* mutations causing a null allele to induce haploinsufficiency and are the most common type of mutations that result in dominant OI (Ben Amor et al., 2013). The most common structural defects in type I collagen causing OI are substitutions for glycine residues in the helical domain and result in moderate to severe, and perinatally lethal OI (Marini et al., 2007; Rauch et al., 2010). In the cells, transcripts containing a premature stop codon triggers the nonsense-mediated mRNA decay (NMD) (Dyle et al., 2020). The p.Q1280Pfs^∗^51 leads to frameshifts and premature termination codons located > 55 bp from the start of last exon of *COL1A1* that is predicted to be targeted by NMD. Hence, no truncated COL1A1 protein will be generated. As compared to missense mutations that result in expressed mutant COL1A1 proteins, frameshift mutations usually cause milder OI phenotypes due to haploinsufficiency of type 1 collagen α, which is the typical mechanism of OI type I (Wang et al., 2015).

Another co-occurrence of mutations was found in P6, who had a heterozygous *COL1A1* c.1668delT (p.G557Vfs^∗^23) and *WNT1* c.677C > T (p.S226L). This may explain that P12 had a mild OI phenotype, due to the presence of frameshift COL1A1 mutation, but severe osteoporosis. The c.1668delT (p.G557Vfs^∗^23) mutation also locates > 55 bp from the start of the last exon of *COL1A1* and therefore is predicted to be targeted by NMD. Indeed, read with *COL1A1* c.1668delT was not found in the RNA sequencing. The mutation, *WNT1* c.677C > T (p.S226L), was expressed in the patient, has been recently indicated in severe recessively-inherited OI phenotypes in patients with homozygous mutations or compound heterozygous mutations (Lu et al., 2018). Modeling of the three-dimensional molecular structure of mutant proteins indicated possible changes in the binding cavity conferred by the p.S226L substitution, while functional *in vitro* studies also supported WNT signaling reduction in cells transfected with p.S226L variant (Lu et al., 2018). In the canonical pathway, WNT binding enables cytosolic β-catenin to escape proteasomal destruction and be translocated into the nucleus, where it activates expression of several genes implicated in bone formation (Baron and Kneissel, 2013). *WNT1* is one of the key ligands to the WNT pathway in bone. Family segregation analysis found that heterozygous *WNT1* mutations lead to autosomal dominant osteoporosis, while homozygous mutations, a more severe OI (Fahiminiya et al., 2013; Laine et al., 2013; Pyott et al., 2013). As reported previously, our patient also presented early onset osteoporosis and kyphotic deformity because of vertebral compression (Makitie et al., 2019). Although the extended family members were not recruited in this present study, we expected that the *WNT1* mutation (c.677C > T) might exist in the local population as its compatibility with life in affected individuals. Further research may be required to understand the clinical impacts on heterozygous carriers and allele frequency in osteoporotic patients due to the founder effect.

Clinically, the presentations of the three patients found to have mutations in *COL2A1* were more compatible with SEDC (p.G654S and p.G441A) and SEMD (p.G1041S) in that they were short in stature with a marked spinal kyphotic curvature. The alpha-1 chain of type II collagen encoded by *COL2A1* has three domains: an N-propeptide which may be involved in the regulation of primary fibril diameters, a triple-helical domain which contains 330 Gly-X-Y repeats and is the predominant motif and a C-propeptide which is considered to be an important factor in the initiation of triple helix formation (Gelse et al., 2003). Previous literature summarized that Glycine to Serine substitution in the triple helical domain of the type 2 procollagen chain is the most common mutation in *COL2A1* (Nishimura et al., 2005). In our cohort, two of the mutations were Glycine to Serine substation but none of them were located in the helical domain. As the mutant protein was predicted to have deleterious effects in protein function, we assumed that these mutations still altered the structural stability of mutant protein and thus manifested negative dominant effects in affected individuals. The recurrent mutation c.3121G > A (p.G1041S) has been reported in 4 individuals listed in the LOVD database^15^. Aligned with previously reported cases (Meredith et al., 2007; Zhang et al., 2015), our patient presented a marked kypholordotic curve along with platyspondyly, a mild pectus carinatum, short lone bones, with dappled metaphyses. However, there was no abnormal ocular problem in this case.

## Conclusion

In conclusion, WES is effective to identify the genetic causes and help differentially diagnosing type I and type II collagenopathies. However, this technique is limited by its inability to detect CNVs because of insufficient uniformity of capturing. Some other genetic techniques, such as MLPA, may be required to complement the diagnostic search for genetic causes. Our case series manifested varied clinical presentations of type I and type II collagenopathies, which highlighting diversity in its genetic causes and potential existence of concurrent mutations either in the same or different genes. Knowing the genetic factors help understand the underlying mechanisms and provide further information for specific treatment and proper genetic counseling for patients and family.

## Data Availability Statement

The raw data supporting the conclusions of this article will be made available by the authors, without undue reservation.

## Ethics Statement

The studies involving human participants were reviewed and approved by the Institutional Review Board of the Cheng Kung University Hospital. Written informed consent to participate in this study was provided by the participants’ legal guardian/next of kin. Written informed consent was obtained from the minor(s)’ legal guardian/next of kin for the publication of any potentially identifiable images or data included in this article.

## Author Contributions

M-CT and Y-YC recruited the patients and collected their clinical information. C-YL, Y-CW, H-WY, and C-HC conducted molecular research. P-CC conducted bioinformatic analysis and supervised the entire research, critically reviewed and revised the manuscript. M-CT and C-HC drafted the manuscript. All authors have read and approved the final version of manuscript.

## Conflict of Interest

The authors declare that the research was conducted in the absence of any commercial or financial relationships that could be construed as a potential conflict of interest.

## Publisher’s Note

All claims expressed in this article are solely those of the authors and do not necessarily represent those of their affiliated organizations, or those of the publisher, the editors and the reviewers. Any product that may be evaluated in this article, or claim that may be made by its manufacturer, is not guaranteed or endorsed by the publisher.

## Footnotes

1
bio-bwa.sourceforge.net

2
www.broadinstitute.org/gatk

3
annovar.openbioinformatics.org/en/latest/

4
gnomad.broadinstitute.org

5
genetics.bwh.harvard.edu/pph2/index.shtml

6
provean.jcvi.org/index.php

7
sift.bii.a-star.edu.sg/

8
www.mutationtaster.org/

9
varseak.bio/

10
daehwankimlab.github.io/hisat2/

11
www-huber.embl.de/HTSeq

12
bioconductor.org/packages/release/bioc/html/DESeq2.html

13
software.broadinstitute.org/gsea/msigdb

14
www.le.ac.uk/ge/collagen

15
https://databases.lovd.nl/shared/genes/COL2A1

## Supplementary Material

Supplementary Figure 1 Co-occurring mutation in *WNT1* in the OI patients. **(A)** Positions of WNT1 variants: TM, transmembrane region. **(B)** Sanger sequencing confirmed WNT1 mutation. **(C)** X-ray films showed marked osteoporosis and thinning of vertebral bodies. **(D)** IGV view of shashimi plot showing reads with *WNT1* c.677C > T (p.S226L) from RNA sequencing of PBMCs of P6.

Supplementary Figure 2 Mutation in *COL2A1* in the SEDC patients. **(A)** Positions of *COL2A1* (NP_001835.3) variants: VWC, von Willebrand factor type C domain; **(C)** Collagen triple helix repeat (20 copies); COLFI, Fibrillar collagen C-terminal domain. **(B–D)** Sanger sequencing confirmed *de novo COL2A1* mutation. **(E–G)** X-ray films showed epiphyseal dysplasia and various degrees of flattening and notching of the vertebral bodies that aligned in kypholordotic curvatures in P7, P8, and P9.
