# Supplementary material for: New Structural and Single Nucleotide Mutations in Type I and Type II Collagens in Taiwanese Children With Type I and Type II Collagenopathies
Source: Front Genet. 2021 Jul 28;12:594285. doi: 10.3389/fgene.2021.594285 (PMC8355745; doi:10.3389/fgene.2021.594285)
Supplement: Supplementary file 1 [file Table_1.docx]

**Supplementary Table 1.** Genetic variants and clinical features found in the patients

| **Gene** | **Patient** | **Variant** | **Inheritance** | **INFO** | **Prediction** | **Height** | **BMD** |
| --- | --- | --- | --- | --- | --- | --- | --- |
| COL1A2 | P1 | c.1-1677_133-441del | Familial | Novel | NA | -7.60 SDS | -5.70 SDS |
|  | P2 | c.1-1677_133-441del | Familial | Novel | NA | -3.67 SDS | -3.1 SDS |
| COL1A1 | P3 | c.3893C>A (p.T1298N) | De novo | Known  (AF=0) | PolyPhen2: benign  SIFT: damaging  PROVEAN: deleterious  Mutation Taster: disease causing | -3.26 SDS | -4.52 SDS |
|  | P4 | c.3893C>A (p.T1298N) | De novo | Known  (AF=0) | PolyPhen2: benign  SIFT: damaging  PROVEAN: deleterious  Mutation Taster: disease causing | -3.88 SDS | -4.66 SDS |
|  | P5 | c.3839_3841delAAGinsCA (p.Q1280Pfs*51) | Familial | Novel | Mutation Taster: disease causing | -2.60 SDS | -1.00 SDS |
|  | P6 | c.1668delT (p.G557Vfs*23) | NA | Known  (AF=0) | varSEAK: no splicing effect | -2.50 SDS | -2.90 SDS |
| COL2A1 | P7 | c.3121G>A (p.G1041S) | De novo | Known  (AF=0) | PolyPhen2: probably damaging  SIFT: damaging  PROVEAN: deleterious  Mutation Taster: disease causing | -6.50 SDS | -3.30 SDS |
|  | P8 | c.1960G>A (p.G654S) | De novo | Novel | PolyPhen2: probably damaging  SIFT: damaging  PROVEAN: deleterious  Mutation Taster: disease causing | -9.30 SDS | -3.60 SDS |
|  | P9 | c.1322C>G (p.G441A) | De novo | Novel | PolyPhen2: probably damaging  SIFT: damaging  PROVEAN: deleterious  Mutation Taster: disease causing | -5.80 SDS | NA |

SDS, standard deviation score; BMD, bone mineral density; NA, not available
