# Supplementary material for: New Structural and Single Nucleotide Mutations in Type I and Type II Collagens in Taiwanese Children With Type I and Type II Collagenopathies
Source: Front Genet. 2021 Jul 28;12:594285. doi: 10.3389/fgene.2021.594285 (PMC8355745; doi:10.3389/fgene.2021.594285)

**Supplementary Table 2.** Primer sequences and amplification information for *COL1A2* exon 1-4 deletion confirmation by PCR and qPCR.


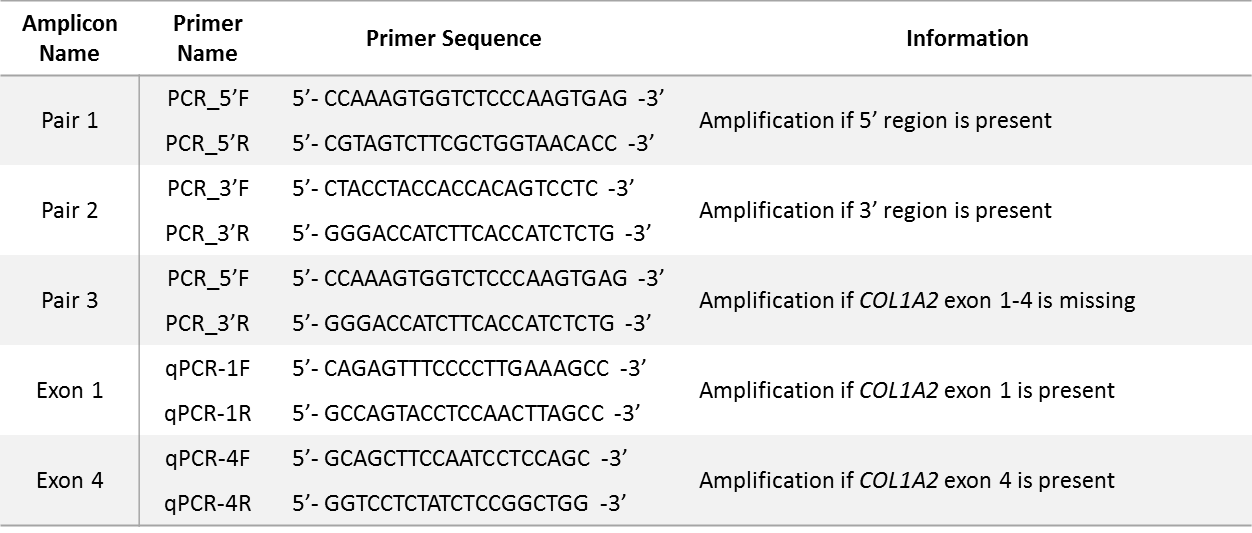

Supplement: Supplementary file 2 [file Table_2.docx]
