# Supplementary material for: New Structural and Single Nucleotide Mutations in Type I and Type II Collagens in Taiwanese Children With Type I and Type II Collagenopathies
Source: Front Genet. 2021 Jul 28;12:594285. doi: 10.3389/fgene.2021.594285 (PMC8355745; doi:10.3389/fgene.2021.594285)
Supplement: Supplementary file 3 [file Table_3.docx]

**Supplementary Table 3.** Rare variants in OI-associated genes in the P1 and P2 family

| **Patient** | **Variant** | **Inheritance** | **INFO** | **Prediction** |
| --- | --- | --- | --- | --- |
| P1 | **COL1A2** c.1-1677_133-441del | Paternal | Novel | NA |
|  | **LRP5** c.2318+2T>C (NM_002335) | Maternal | Novel | varSEAK: exon skipping  Mutation Taster: disease causing |
| P2 | **COL1A2** c.1-1677_133-441del | Paternal | Novel | NA |
|  | **LRP5** c.2318+2T>C (NM_002335) | Maternal | Novel | varSEAK: exon skipping  Mutation Taster: disease causing |
| Father | **COL1A2** c.1-1677_133-441del | Familial | Novel | NA |
| Mother | **LRP5** c.2318+2T>C (NM_002335) | Unknown | Novel | varSEAK: exon skipping  Mutation Taster: disease causing |

AF, allele frequency; NA, not avaliable.
