# Supplementary figures and images for: New Structural and Single Nucleotide Mutations in Type I and Type II Collagens in Taiwanese Children With Type I and Type II Collagenopathies
Source: Front Genet. 2021 Jul 28;12:594285. doi: 10.3389/fgene.2021.594285 (PMC8355745; doi:10.3389/fgene.2021.594285)

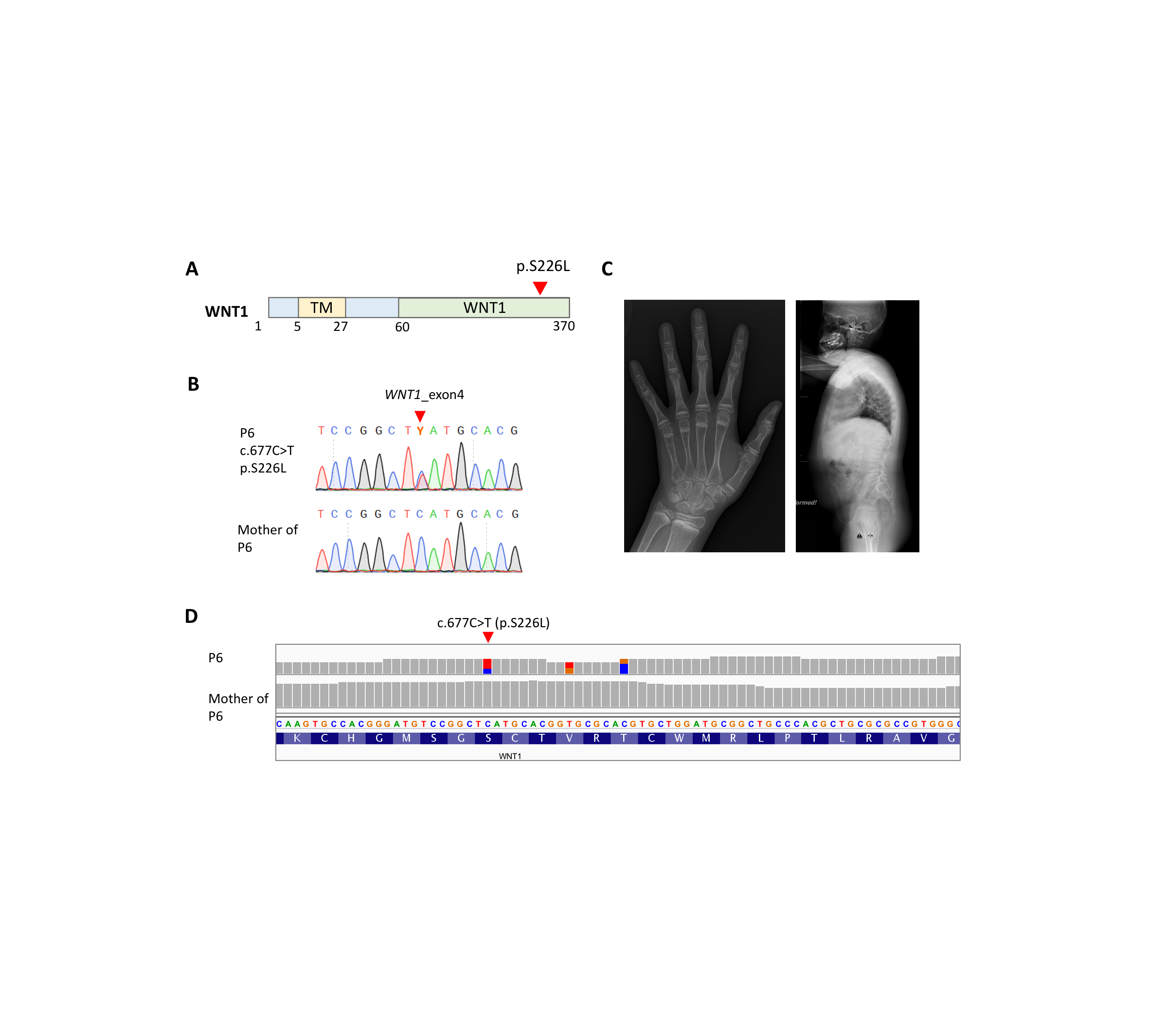

Supplement: Supplementary Figure 1 — Co-occurring mutation in WNT1 in the OI patients. (A) Positions of WNT1 variants: TM, transmembrane region. (B) Sanger sequencing confirmed WNT1 mutation. (C) X-ray films showed marked osteoporosis and thinning of vertebral bodies. (D) IGV view of shashimi plot showing reads with WNT1 c.677C > T (p.S226L) from RNA sequencing of PBMCs of P6. [file Image_1.TIF]

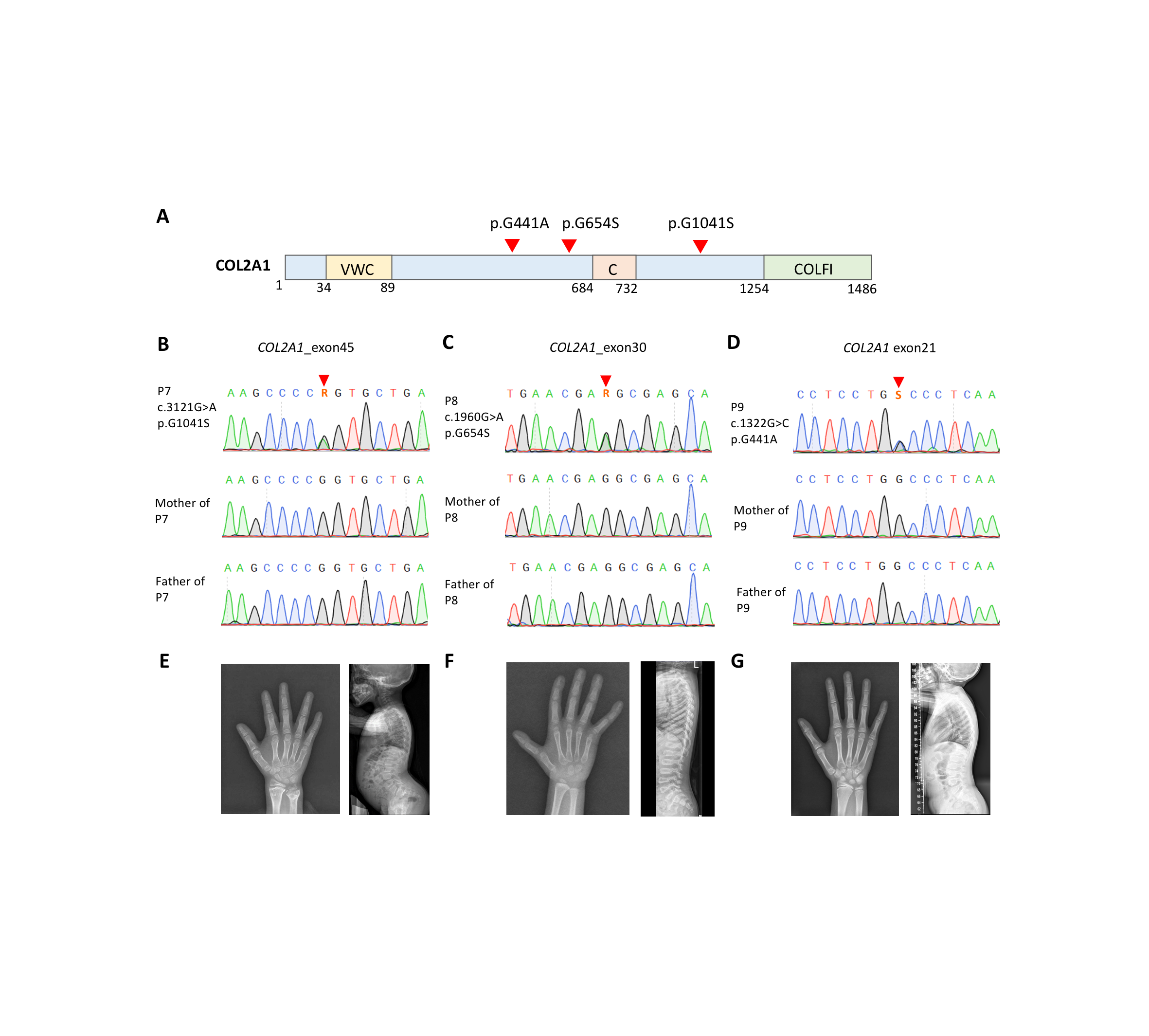

Supplement: Supplementary Figure 2 — Mutation in COL2A1 in the SEDC patients. (A) Positions of COL2A1 (NP_001835.3) variants: VWC, von Willebrand factor type C domain; (C) Collagen triple helix repeat (20 copies); COLFI, Fibrillar collagen C-terminal domain. (B–D) Sanger sequencing confirmed de novo COL2A1 mutation. (E–G) X-ray films showed epiphyseal dysplasia and various degrees of flattening and notching of the vertebral bodies that aligned in kypholordotic curvatures in P7, P8, and P9. [file Image_2.TIF]
